# Muscle fat infiltration following whiplash: A computed tomography and magnetic resonance imaging comparison

**DOI:** 10.1371/journal.pone.0234061

**Published:** 2020-06-02

**Authors:** James M. Elliott, Andrew C. Smith, Mark A. Hoggarth, Stephanie R. Albin, Ken A. Weber, Mat Haager, Joel Fundaun, Marie Wasielewski, D. Mark Courtney, Todd B. Parrish

**Affiliations:** 1 Discipline of Physiotherapy, Faculty of Medicine and Health, The University of Sydney, & The Northern Sydney Local Health District, The Kolling Research Institute, St. Leonards, New South Wales, Australia; 2 Physical Therapy and Human Movement Sciences, Feinberg School of Medicine, Northwestern University, Chicago, Illinois, United States of America; 3 School of Physical Therapy, Regis University, Denver, CO, United States of America; 4 Department of Biomedical Engineering, Northwestern University, Evanston, Illinois, United States of America; 5 Department of Anesthesiology, Perioperative and Pain Medicine, Systems Neuroscience and Pain Lab, Stanford University, Palo Alto, California, United States of America; 6 Department of Emergency Medicine Feinberg School of Medicine, Northwestern University, Chicago, Illinois, United States of America; 7 Department of Radiology, Feinberg School of Medicine, Northwestern University, Chicago, Illinois, United States of America; McLean Hospital, UNITED STATES

## Abstract

Here we present a secondary analysis from a parent database of 97 acutely injured participants enrolled in a prospective inception cohort study of whiplash recovery after motor vehicle collision (MVC). The purpose was to investigate the deep and superficial neck extensor muscles with peri-traumatic computed tomography (CT) and longitudinal measures of magnetic resonance imaging (MRI) in participants with varying levels of whiplash-related disability. Thirty-six underwent standard care imaging of the cervical spine with CT at a level-1 trauma designated emergency department. All 36 participants were assessed with MRI of the cervical spine at <1-week, 2-weeks, 3-, and 12-months post-injury and classified into three groups using initial pain severity and percentage scores on the Neck Disability Index (recovered (NDI of 0–8%), mild (NDI of 10–28%), or severe (NDI ≥ 30%)) at 3-months post MVC. CT muscle attenuation values were significantly correlated to muscle fat infiltration (MFI) on MRI at one-week post MVC. There was no significant difference in muscle attenuation across groups at the time of enrollment. A trend of lower muscle attenuation in the deep compared to the superficial extensors was observed in the severe group. MFI values in the deep muscles on MRI were significantly higher in the severe group when compared to the mild group at 1-year post MVC. This study provides further evidence that the magnitude of 1) deep MFI appears unique to those at risk of and eventually transitioning to chronic WAD and that 2) pre- or peri-traumatic muscular health, determined by CT muscle attenuation, may be contribute to our understanding of long-term recovery.

## Introduction

Neck pain arising from a motor vehicle collision (MVC) can significantly influence quality of life for nearly half of those exposed to and injured from such an event. [1] The term whiplash arises from the osteokinematic observations of rapid multi-planar acceleration/deceleration of the head and neck, [2] whereby a spectrum of tissue loading has been reported in cadavers, animal species, and simulation. [3] These range from no apparent damage to strains beyond physiological limits in the facet joints, [4, 5] partial ruptures of the facet capsule, [6] or damage involving ligaments, [7] arteries, [8] and the intervertebral disc. [9]

The subsequent multifactorial clinical presentations, which are commonly referred to as whiplash-associated disorders (WAD), [10] include, but are not limited to, neck-related disability, [11] sensorimotor disturbances, [12] distress, [13] and neck muscle degeneration and weakness. [14–17] The personal, public, and socioeconomic burdens of WAD are complex [18] with inconsistent scientific explanations [19] and a lack of strong evidence to mitigate the persistent pain, disability, and loss of function for the millions affected worldwide. [20]

Those injured and experiencing persistent pain, disability, and loss of function may benefit if findings from advanced diagnostic imaging studies were identified, related to the clinical signs and symptoms, and able to inform a plan of care. However, no consistent relationship between MVC-related pathology and the clinical course has been observed with conventional imaging, leading to increased scepticism around the value of imaging findings. [21–23] However, one consistent magnetic resonance imaging (MRI) finding in whiplash appears interesting; that of neck muscle fat infiltration (MFI), which i) has shown to be present in those with chronic whiplash in both the deep and superficial neck muscles, [16, 24, 25] ii) appears to be partisan to those with traumatic neck disorders, [26, 27] iii) is expressed to a larger magnitude in those transitioning to chronic WAD-related disability, [14, 28] and iv) has been reported across three different countries with different insurance schemas (Sweden, [15] Australia, [14] and the United States [28, 29]).

Non-invasive quantification of muscle fat can be achieved with a number of imaging modalities, such as computed tomography (CT) [30] and MRI. [31, 32] Water and fat MRI, derived from multi-echo acquisitions (e.g. Dixon), provide for a robust measure [33] of tracking longitudinal changes to paraspinal muscle composition characterized by muscle fat. [34, 35] While such changes are interesting and show potential prognostic value, [28] it is difficult to determine if these muscle changes are the cause or result of pain onset following injury. In particular, does trauma to the head/neck (e.g. whiplash) cause such changes? Do other confounders like age, [36–39] sex, [39] body composition, [38, 40] physical activity levels, [41] pain duration, [42, 43] or do co-occurring, [44, 45] or pre-collision overall health, [46] influence muscle composition?

Furthermore, while MRI measures of MFI have shown to be related to the severity of whiplash, referral for MR imaging in the acute stage is not considered to be ‘usually appropriate’ per available guidelines of suspected spine trauma unless there is neurological involvement or overt ligamentous injury is suspected. [47–49] In the absence of overt cervical trauma or neurological deficits, CT is the preferred initial imaging modality given the primary concern for fracture. CT can also be used to evaluate the composition of muscle, but whether or not standard-of-care CT measures of muscle fat (determined by lower radiation attenuation) are also related to MRI findings of neck MFI and the clinical course of whiplash is currently unknown. The primary aim of this prospective study was to determine if peri-traumatic neck muscle attenuation values from standard emergent care CT scans were correlated with higher expressions of muscle fat on MRI within one-week post-MVC (primary aim 1a) and if these CT measures were related to severity-group differences one-year after whiplash injury (primary aim 1b). A secondary aim was to determine if the expressions of fat were different across the multi-layered deep and superficial muscles traversing the cervical region and if they related to the heterogeneity of WAD recovery. A tertiary aim was to assess differences in MFI (as measured by MRI) between those that recovered versus those that did not over a one-year time period (for the deep neck extensors, superficial neck muscles), and to see if MFI at one-year post-MVC was related to clinical outcomes of disability and pain intensity.

## Materials and methods

This is a secondary analysis of data drawn from a prospective study investigating the neuromuscular mechanisms underlying poor recovery following an MVC-related whiplash injury (ClinicalTrials.gov Identifier: NCT02157038). Ninety-seven participants were recruited, consented, and enrolled via an urban academic emergency medicine department and were eligible provided they both reported MVC-related neck pain (4 or > on a numeric pain rating scale) and were within the Quebec Task Force Classification category of WAD Grade II (movement restriction with no radicular symptoms). [10] Furthermore, participants were eligible for this study provided they had sufficient probability of injury as predicted by either the Canadian Cervical Spine Rule (CCR) or National Emergency X-Ray Utilization Study Low Risk Rule (NEXUS) [50] criteria to warrant a CT scan of their cervical spine. As part of the longitudinal parent study, all enrolled participants (whether they had a CT scan or not), underwent serial MRI examination at < 1 week, 2-weeks, 3-months, and 12-months post injury to quantify MFI in select bodily muscles. All participants completed a suite of questionnaires capturing neck-related interference, hyperarousal, anxiety, depression, and a performed a motor task to quantify maximal volitional plantar flexor torques (not reported in this sub-study).

Exclusion criteria were those participants younger than 18 or older than 65 years of age, one or more previous MVC’s in their lifetime, treatment for neck pain disorders in the past ten years, any nervous system disorder (e.g. stroke, Parkinson’s), metabolic system disorder (e.g. diabetes), or those who, by standard emergency medical services’ protocols were deemed to be at risk for multi-system trauma. The Institutional Review Board of Northwestern University, Feinberg School of Medicine granted approval (STU00090769) and all participants provided informed written consent.

### Clinical outcomes

#### CT study

Clinically indicated cervical spine CT imaging (SOMATOM Force, Siemens, Erlangen, Germany)—without contrast—was performed at the emergency medicine department. 2.5mm helical images were obtained through the entire cervical spine, ranging from 70–125 slices, kVp 100, reference mAs 335/300, detector collimation 0.6mm, and CT dose index volume (CTDIvol) of 22.6L (mGy). Bone, soft-tissues, and the 2D coronal and sagittal reconstructed images were reviewed and approved by board-certified neuroradiologists with specific training in spine imaging. Defined regions of interest (ROIs) were manually traced bilaterally over the following cervical muscles: multifidii, semispinalis cervicis, splenius capitis, and sternocleidomastoids (SCMs) from C3-C7 on the CT scans. This was performed by a board-certified orthopaedic resident of physical therapy (JF) with clinical specialization in cervical spine disorders and research experience in manual segmentation of paraspinal muscles on CT and MRI. The deep neck extensor group included the multifidii and semispinalis cervicis (combined for measurement). The superficial neck group included the splenius capitis and the SCMs.

#### CT muscle fat analysis

**Fig 1:**
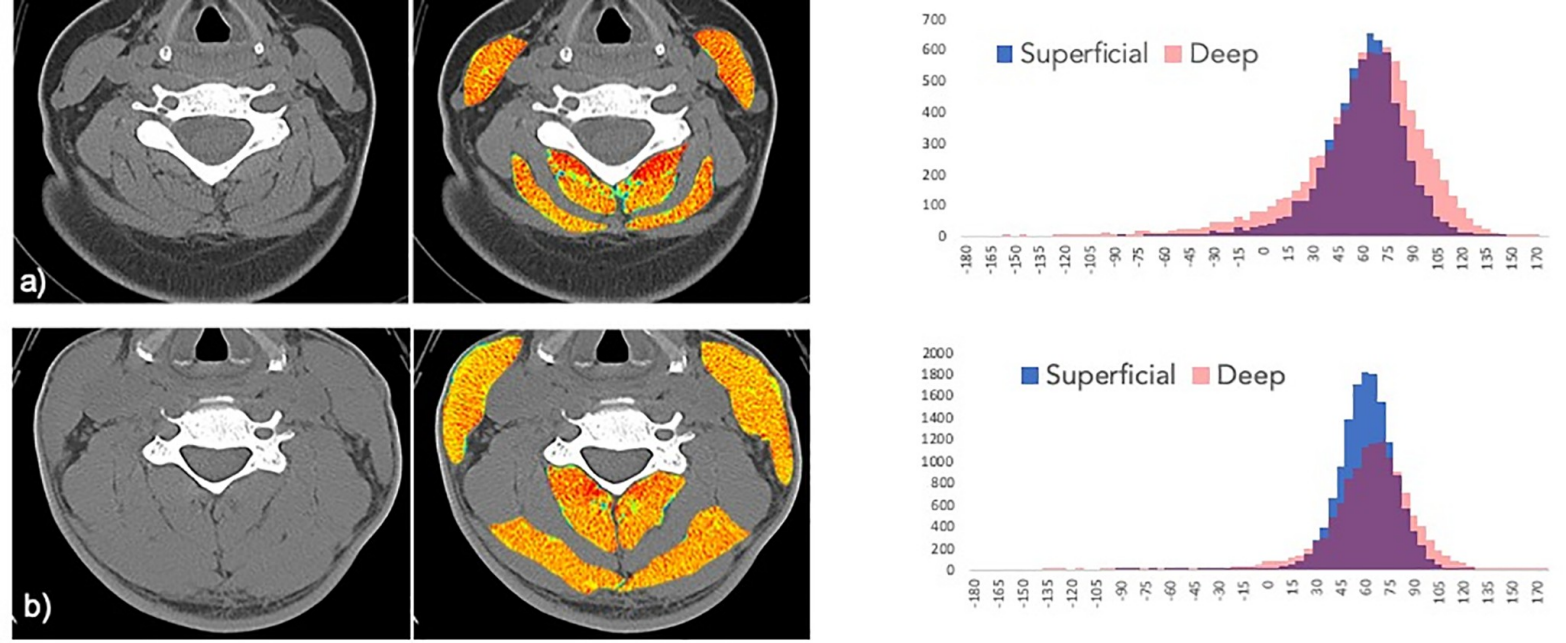
Radiation attenuation map of neck muscles at C5. ** a)** Subject 1 is a 32-year- old female with a body mass index of 25.8 kg m^2^ with poor recovery at 12-months post-MVC. The paraspinal muscles exhibit extensive visible fat within the fascia surrounding skeletal muscle making up 5.1% of total tissue area. Exclusive of the intermuscular fat, the mean overall radiation attenuation is 53.9 HU. **b)** Subject 2 is a 50-year-old male with a body mass index 27.5 kg m^2^ reporting full-recovery at 12-months post-MVC. There is much less visible regions of intermuscular fat infiltration (light blue) comprising 1.2% of total area, a value on the order of 4 fold lower than Subject 1. Exclusive of the macroscopic fat infiltration, the muscles show an overall mean attenuation of 59.0 HU. Corresponding histograms display the HU ranges and counts for the deep (pink) and superficial (blue) musculature (purple represents the HU overlap between the deep and superficial muscles).

A Hounsfield unit (HU) analysis using predefined HU ranges demarcating adipose tissue (usually -190 to -30 HU) and muscle tissue (usually -29 HU to +150 HU) was used to characterize muscle tissue based on radiation attenuation values between -190 and +150 HUs). [51] Fig 1a and 1b was created using ROIs drawn manually in the Matlab programming environment. HU values were recorded from groupings of the respective deep and superficial muscles. Histograms were created with a bin width of 10, from -160 HU to +160 HU.

#### MRI study

All post-MVC MRI data were collected with a 3.0T Prisma scanner (Siemens, Erlangen, Germany). A localizer scan and a T2-weighted sagittal turbo spin echo sequence was performed to determine the location of the fat-water scan.

#### MRI muscle fat analysis

High-resolution 3D fat-water images of the cervical were acquired using a dual-echo gradient-echo sequence (2-point Dixon, TR = 7.05 ms, TE1 = 2.46 ms, TE2 = 3.69 ms, flip angle = 12°, bandwidth = 510 Hz/pixel, FOV = 190 x 320 mm^2^, slab oversampling of 20% with 40 partitions to prevent aliasing in the anterior-posterior direction, in-plane resolution = 0.7 x 0.7 mm^2^, slice thickness = 3.0 mm, number of averages = 6, acquisition time = 4min 5s). A 64-channel head/neck coil was used as a receiver coil to improve signal-to-noise. This scan covered the cephalad portion of C3 through the caudal portion of the C7 vertebral end plate.

#### Muscle water-fat quantification

**Fig 2:**
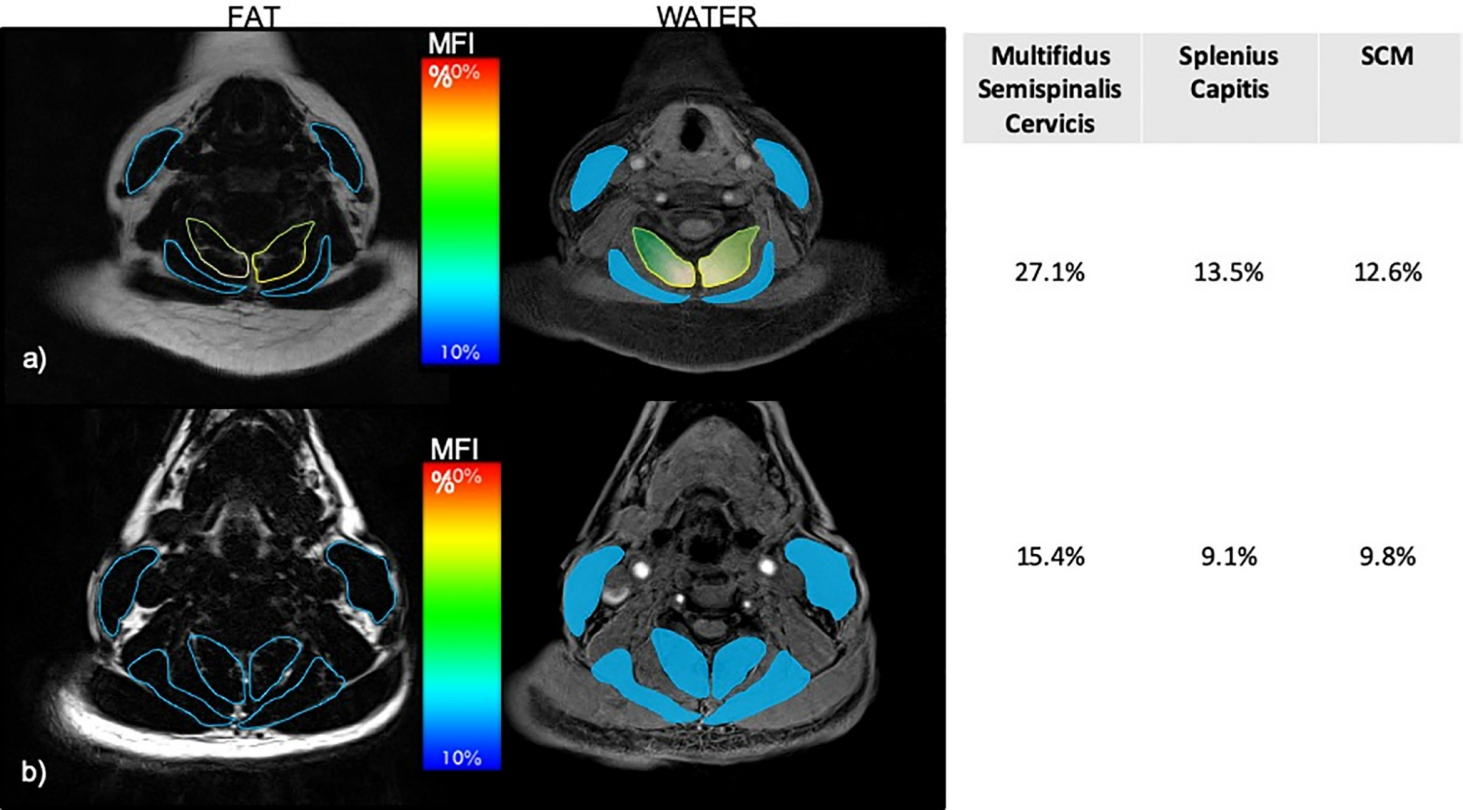
Fat/water MRI of the deep and superficial neck muscles at C5 within one-week of the MVC. **a)** represents the 32-year-old female with poor recovery at 12-months post-MVC with corresponding MFI values (%). **b)** represents the 50-year-old male reporting full-recovery at 12-months post-MVC with corresponding MFI values (%*)*.

Defined regions of interest (ROIs) were manually traced over each of the bilateral cervical muscles from C3-C7 on the water-fat images. [32] The deep and superficial neck muscle groups were defined in the same fashion as the CT study (above) (Fig 2).

The MFI (%) from 3D water-fat imaging was created from the mean pixel intensity of fat-only (Fat) and the mean pixel intensity of water-only (Water) images with the following equation:

MFI(%)=Fat/(Fat+Water)*100

### Subjective (self-reported) clinical outcomes

#### Self-reported neck-related disability

Self-reported neck-related disability was measured using the Neck Disability Index (NDI), which has been used extensively to quantify neck disability. [52] Percentage scores ≥ 30% have been reported to indicate moderate/severe neck-related disability. [28] Previous literature has shown that recovery occurs for a substantial proportion of patients in the initial 3 months after the MVC, but recovery rates level off and show little improvement after 3 months. [53]

#### The numeric pain rating scale (NPRS)

Is a self-report unidimensional measure of pain intensity in which the respondent selects a whole number (0–10 integers) that best reflects the intensity of their pain. [11] Higher initial pain (> 5.5/10) intensity has been associated with worse outcomes. [11]

Accordingly, participants were classified as severe based on an initial NPRS score of ≥ 5/10 in tandem with an NDI score of ≥ 30% at 3 months (the other two groups were classified 0–8%, recovered, 10–28%, mild).

### Statistical analyses

All analyses were performed using SPSS Version 23.0 statistical software (IBM Corporation, Armonk, NY). Baseline descriptive statistics were summarized and assessed for potentially important differences. Pearson correlations were used to establish relationships between CT and MRI measures of MFI, for both the deep and superficial muscles (primary aim 1a). Peri-traumatic CTs between the three groups were compared with analysis of covariance (ANCOVA) for the deep, superficial muscles with age as a covariate (primary aim 1b). For MRI measures of MFI, changes across time for the deep and the superficial muscles were assessed using linear mixed modelling. Group, time and group-by-time interactions were modelled as fixed effects, and MFI was estimated using separate, random-intercept, and slope linear mixed models. Baseline scores and age were used as covariates in the model. The primary analysis of interest was the adjusted pairwise comparison of each muscle group at the one-year follow-up (secondary aim). In the severe group, planned comparisons of the deep and superficial muscles using MRI at all four time points were performed with paired t-tests (secondary aim). Lastly, Pearson correlations were used to establish relationships of MRI measures of MFI at the one-year timepoint versus both the NDI and the NPRS (tertiary aim).

## Results and discussion

**Fig 3:**
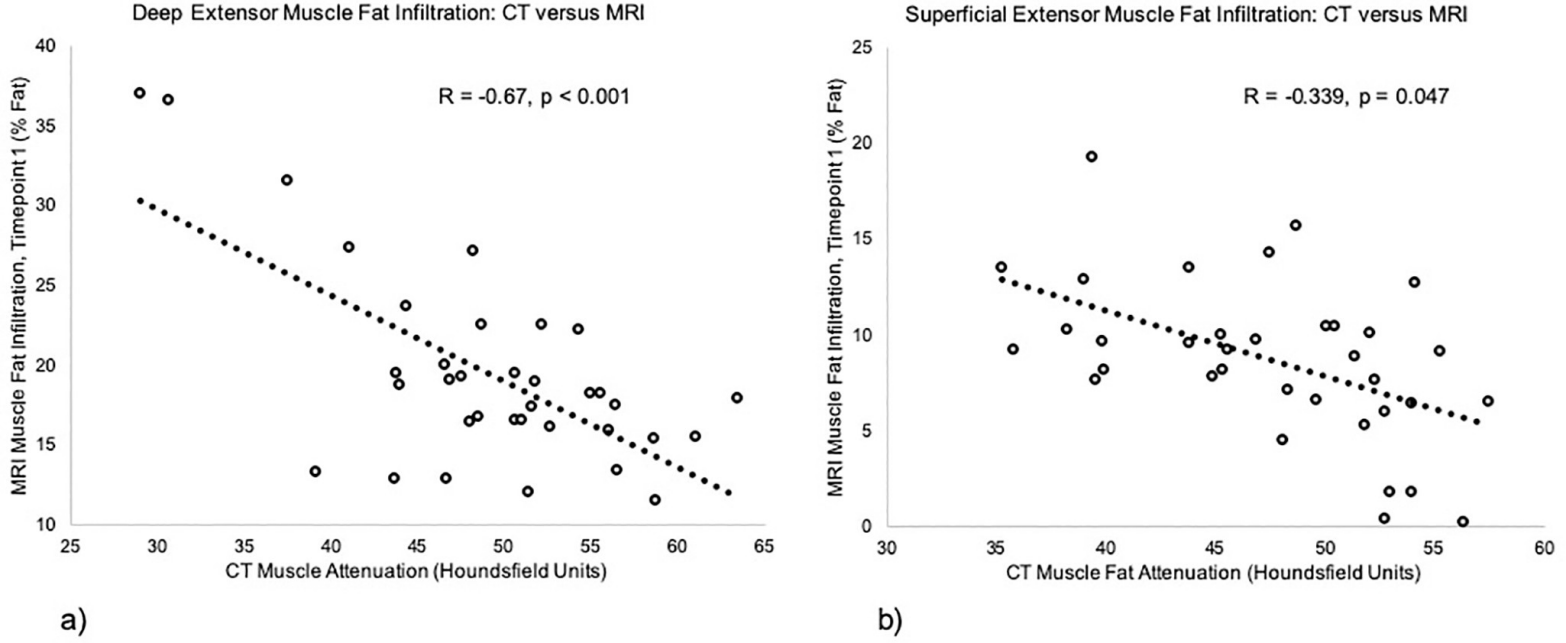
Correlations between. **a)** deep and **b)** superficial muscle attenuation from emergent care CT and < 1-week MRI measures of MFI.

The demographics of all participants are displayed in Table 1 (with 13/36 (36%) recovered; 12/36 (33%) mild; 11/36 (31%) severe). Muscle attenuation from emergent care CT (Table 2) and the within < 1-week MRI measures of MFI were significantly correlated, for both the deep (R = -0.67, p < 0.001) and superficial groups (R = -0.34, p = 0.047) (Fig 3a and 3b) (primary aim 1a).

**Table 1:** Age, gender, and demographics of subject groups. Data displayed as mean (SD) except gender (%).

	**Recovered (n = 13)**	**Mild (n = 12)**	**Severe (n = 11)**
**Age (years)**	34.5 (10.4)	35.6 (12.5)	35.2 (12.2)
Gender (n, % Female)**	84.6	100.0	72.7
**BMI (Kg/m**^**2**^**)**	24.6 (3.9)	23.1 (3.8)	25.5 (5.6)
**< 1 week of MVC (t1)**	**Recovered (n = 13)**	**Mild (n = 12)**	**Severe (n = 11)**
**NDI(%)**	30.5 (12.6)	38.2 (15.9)	49.5 (13.6)
**Pain Intensity (NPRS)**	3.9 (2.3)	5.3 (2.2)	7.7 (1.7)
**2 weeks after MVC (t2)**	**Recovered (n = 13)**	**Mild (n = 12)**	**Severe (n = 11)**
**NDI (%)**	25.8 (16.6)	30.3 (11.6)	47.5 (12.0)
**Pain Intensity (NPRS)**	3.5 (2.1)	5.1 (2.2)	7.0 (2.0)
**3 months after MVC (t3)**	**Recovered (n = 13)**	**Mild (n = 12)**	**Severe (n = 11)**
**NDI (%)**	5.2 (4.9)	21.3 (9.9)	39.7 (11.6)
**Pain Intensity (NPRS)**	1.2 (1.1)	3.6 (2.0)	4.2 (3.0)
**12 months after MVC (t4)**	**Recovered (n = 13)**	**Mild (n = 12)**	**Severe (n = 11)**
**NDI (%)**	8.6 (9.9)	20.0 (6.5)	23.2 (16.9)
**Pain Intensity (NPRS)**	1.6 (2.2)	3.9 (2.3)	4.1 (3.8)

BMI: Body Mass Index

NDI: Neck Disability Index

MVC: Motor Vehicle Collision

NRPS: Numeric Pain Rating Scale

t1: time point 1

t2: time point 2

t3: time point 3

t4: time point 4

** 86.1% (31/36) of our cohort were Female

**Table 2:** Computed tomography radiation attenuation values for deep and superficial neck muscles in groups at the time of emergency department visit. Data displayed as Hounsfield Units mean (SD).

Emergent care CT	Recovered (n = 13)	Mild (n = 12)	Severe (n = 11)
**Deep**	52.5 (7.0)	48.5 (7.9)	47.1 (7.7)
**Superficial**	49.2 (6.4)	46.4 (5.7)	47.4 (6.7)

The peri-traumatic CT measure of muscle attenuation, based on HUs, in the deep muscles showed a trend of being worse (lower attenuation) in the severe group compared to the recovered group (mean difference 5.18 HU, 95% CI: 0.23 to 10.58, p = 0.06 (primary aim 1b). However, there was no group differences for muscle attenuation measured by CT for superficial neck muscles (p = 0.57) or for the SCMs (p = 0.65). The deep muscles had significantly greater MFI on MRI at all timepoints compared to the superficial muscles (p < .001) (Table 3) (secondary aim). There were no significant group by time interactions for the deep or superficial neck muscles as measured by MRI in this subsample (n of 36) of the larger prospective study (n of 97). However, there were significant pairwise comparisons for the deep neck muscles with the severe group exhibiting greater MFI at 1-year compared to the recovered group (p < 0.05). For the deep muscles, the severe group had larger amounts of MRI-measured MFI at 1-year post-MVC (p < 001). MFI of the deep muscles on MRI at one year was significantly correlated with NDI scores (R = 0.43, p = 0.02) and showed a trend for NPRS (R = 0.33, p = 0.08) (tertiary aim).

**Table 3:** Magnetic resonance MFI % in groups across all 4 time points. Data displayed as mean (SD).

**< 1 week of MVC (t1)**	**Recovered (n = 13)**	**Mild (n = 12)**	**Severe (n = 11)**
**Deep**	17.5 (4.2)	19.8 (7.5)	21.1 (6.5)
**Superficial**	7.9 (3.8)	10.7 (4.2)	7.6 (4.1)
**2 weeks after MVC (t2)**	**Recovered (n = 13)**	**Mild (n = 12)**	**Severe (n = 11)**
**Deep**	17.3 (4.0)	19.5 (7.0)	21.6 (5.9)
**Superficial**	7.7 (4.2)	9.1 (3.2)	7.7 (3.6)
**3 months after MVC (t3)**	**Recovered (n = 13)**	**Mild (n = 12)**	**Severe (n = 11)**
**Deep**	17.8 (4.7)	19.1 (6.7)	22.1 (6.1)
**Superficial**	8.4 (5.2)	9.8 (3.2)	7.3 (3.2)
**12 months after MVC (t4)**	**Recovered (n = 13)**	**Mild (n = 12)**	**Severe (n = 11)**
**Deep**	17.0 (5.3)	19.9 (7.8)	22.2 (6.2)
**Superficial**	7.0 (3.5)	9.6 (3.4)	10.0 (3.2)

MFI: Muscle Fat Infiltration

MVC: Motor Vehicle Collision

t1: time point 1

t2: time point 2

t3: time point 3

t4: time point 4

Consistent with previous investigations, [14–16, 28] this study provides further evidence for the presence of neck muscle fat in participants with varying levels of neck-related interference following MVC-related whiplash injury. Unique to this study was the observed trend of reduced attenuation values for the deep neck muscles (suggestive of greater adipose tissue distribution and less likely to be characteristic of healthy muscle) [54] on clinically warranted CT scans in the group of participants who later demonstrated a worse outcome at 1-year post-MVC. The severe group also exhibited significantly greater muscle fat in the deep muscles at 1-year post-MVC with MRI when compared to those reporting milder pain. This contrasted our findings where no appreciable group differences in the superficial muscle MFI percentages were identified at any time point on MRI, suggesting composition of the deep cervical muscles may contribute to overall cervical spine health, and represent a biomarker for susceptibility of chronic WAD. It is plausible the deep extensors may contain more intermuscular fat than the superficial muscles. Accordingly, the higher MFI in the deep muscles (multifidus and semispinalis cervicis) may be the consequence of poorer muscle quality that is realized when segmenting them together. Future work, using higher resolution MRI, should aim to segment the two deeper muscles in isolation and consider the spatial distribution in tandem with the magnitude of MFI. [55, 56]

Previous work using T1-weighted and fat/water imaging demonstrated greater expressions of MFI over time, occurring in the deep muscles between 1–2 weeks [28] and in all of the neck muscles at 1-month post injury. [14] This particular study also identified larger magnitudes of MFI in the deep muscles on fat/water MRI in those with poor recovery, but this was not featured in all of the neck muscles. These findings do not definitively answer questions around cause-and-effect of MFI in whiplash or their influence on recovery. However, the presence of more inter- and intra-muscular fat in the deeper muscles traversing the cervical spine raises questions as to whether the biological health (e.g. size and composition) of these muscles contributes to a potential phenotypic expression of chronic WAD and if it translates to long-term deficits in motor function specific to the neck or in general, the entire body. [16, 29, 57, 58]

Clinical imaging guidelines, such as the American College of Radiology—Appropriateness Criteria exist and are used to assist providers in making the most appropriate decision with respect to the performance of imaging tests for a specific clinical condition. The criteria for determination of whether imaging is indicated in suspected spine trauma, and the recommended modality are based upon the CCR and the NEXUS criteria. In the presence of positive clinical assessment findings derived from the CCR [59] or the NEXUS, [60] CT is the initial imaging modality determined to be *usually appropriate*. Of interest, only 36 of the 97 enrolled participants (37% of the total cohort) underwent a CT of the cervical spine at the discretion of the treating emergency medicine clinicians who felt the patient had sufficient probability of injury (e.g. midline tenderness, excessive pain, limited mobility) as predicted by either the CCR or NEXUS criteria to warrant imaging. [50] This is not to suggest the remaining 61 participants were not injured, rather the decision to forego imaging was the result of the patient’s negative status on the CCR or NEXUS, and the exam findings from the treating emergency medicine clinician.

While CT (due to its rapid image acquisition and near ubiquitous availability twenty-four hours a day in most emergency medicine departments) is the modality of choice to rule out traumatic fracture or other destabilizing injury, [49] it can also provide for a measure of muscle and fat [54] and pre-existing degenerative pathologies. [61] In our study, the available emergent, and clinically warranted, CT scans were used to determine the peri-traumatic composition of deep and superficial neck muscles using pre-determined radiation attenuation ranges for muscle and fat. [51] Interestingly, our results demonstrate a trend for lower HUs (suggesting less muscle and greater distributions of adipose tissue) in the deeper muscles but not in the superficial muscles, just hours after the crash. (Fig 1a and 1b) This is consistent with the longitudinal MRI findings where MFI of the deeper muscles defined group differences at each time point. And, consistent with the wider whiplash literature, ~31% of the cohort (11/36) followed a poor recovery trajectory, 33% (12/36) reported milder neck-related disability and 36% (13/36) recovered spontaneously.

Reductions in muscle attenuation on CT have shown to be a result of i) older age, [62, 63] ii) obesity, [64, 65] iii) diabetes, [64, 65] iv) cancer, [66, 67] v) degenerative conditions of the spine [68] or peripheral joints. [51, 69] Each of these conditions is associated with reduced attenuation on the order of 3–6 HU, [51] which fits our group mean difference of 5.18 HU with the severe group having lower radiation attenuation of the deep muscles on acute CT. Of note, the Hounsfield attenuation coefficient ranges typically reported in the literature tend to agree that adipose tissue lies between -190 and -30 HU, [54, 70] and that muscle is typically from 0 to somewhere between +100 HU or more. This leaves a gap from -29 to 0 HU that may be considered a ‘transition area’ between muscle and fat, or often considered to be “low attenuation muscle tissue”. [54] Moreover, additional infiltration of adipose tissue within muscle, has shown to decrease the muscle density value below + 50 HU. [54] While all of our participants had neck muscle attenuation values that primarily fell within the HU range consistent with muscle, those with an eventual poor recovery profile presented with lower attenuation values (< + 50 HU) across a wider range compared to those who considered themselves recovered. While the precise mechanisms for these findings are unknown, it is plausible that lower CT muscle attenuation in the aftermath of the MVC may suggest poor pre-collision muscle health or an acute inflammatory response to injury, either of which could yield prognostic value. Controlled prospective cohort studies with larger sample sizes are warranted to determine if peri-traumatic muscle attenuation analyses from standard of care CT scans would add to our mechanistic understanding of muscle compositional changes and whether such changes enhance the prognostic profile of whiplash on a patient-by-patient basis.

We can be confident the findings of lower attenuation in the deep neck muscles at the time of injury are not the result of diabetes, cancer, or previous trauma with neck disorders, as we were careful to screen each participant for such past medical histories or conditions. The effects of age on deep or superficial muscle composition are difficult to evaluate precisely as age-related deconditioning, loss of fat-free muscle mass, reduced strength and endurance could all influence lower muscle attenuation due to atrophy and increased fat mass. This sub study was unable to determine any potential age effect as the mean age across all three groups was similar; Severe (mean age 35.2); recovered (mean age 34.5) and those with milder symptoms (mean age of 35.6) (see Table 1). To our knowledge, little is known about CT muscle attenuation in healthy people across the lifespan or the influence of demographic and anthropometric variables, such as age, sex, body habitus, and ethnicity. [37] Normal age- and sex-related change in muscle composition needs to be established.

While the MRI findings of muscle fat have been widely reported across a number of musculoskeletal conditions (spinal pain, [71, 72] acute to chronic whiplash, [73] rotator cuff pathology, [74] knee osteoarthritis. [75, 76]) and neuromuscular disorders (e.g. Charcot-Marie Tooth neuropathy, [77] Duchenne muscular dystrophy, [78] Pompe disease, [79] facioscapulohumeral dystrophy, [80] spinal muscular atrophy [81], spinal cord injury [82]), clinical practice is not amenable to the serial performance of an expensive and often difficult to access MRI for each and every patient with any of these conditions. This is not to suggest patients receiving care (or clinical practice) “need(s) more imaging” to answer such clinical questions. On the contrary, it is plausible that the warranted cervical spine CT exam at the time of the emergency medicine visit could be used as a measure of pre- or peri-traumatic muscle health to uncover a potential phenotypic expression of chronic WAD. [61] Future prospective work is required.

Another limitation of our study relates to the accuracy of comparing HUs to MFI % from fat/water MRI. While muscle attenuation from emergent care CT was negatively correlated to the within < 1-week MRI measures of MFI, a 1:1 relationship does not exist. It is plausible that MRI measurement errors due to field inhomogeneity, patient characteristics (body habitus) and movement artefact, which limit spatial resolution, could explain the disparities. However, muscle fat infiltration on either CT or MRI continues to emerge as a potential biomarker of systemic, musculoskeletal, and neuromuscular disorders, being linked to reduced strength, impaired physical function, risk for fracture, increased pain, and pathology. [51]

The small number of participants that underwent a CT is both a limitation and a positive finding. It is a limitation in that it can, at its best, only provide a foundation for establishment of a new diagnostic/prognostic landscape for WAD. Accordingly, future prospective studies should aim to include a larger number of participants where CT scan is clinically warranted and can be used to compare muscle attenuation to findings of MFI using advanced MRI. On the contrary, the low number of participants receiving CT scan in this study reflects good clinical practice whereby existing imaging guidelines (CCR and NEXUS) are used to help guide informed clinical decisions, avoiding unnecessary exposure to ionizing radiation.

## Conclusions

Inter- and intra-muscular fat infiltration is a potential cause of reduced attenuation of muscle on CT and this has been featured in participants with limited mobility, obesity, diabetes, cancer, and degenerative conditions of the spine and peripheral joints. Findings from this preliminary work suggests that neck muscle attenuation profiles on clinically warranted CT scans in the peritraumatic stage may be a piece of the clinical picture. Further research is required to determine if and how such muscle profiles contribute to recovery models in whiplash and whether they would contribute towards informing management strategies.

## Supporting information

S1 Data (XLS)
